# Gut-derived biofilm-forming bacteria as a source of catheter-associated infections: inhibitory effects of *O*-alkyl naringenin derivatives

**DOI:** 10.3389/fcimb.2026.1768480

**Published:** 2026-03-13

**Authors:** Anna Duda-Madej, Wojciech Tabor, Szymon Viscardi, Monika Oleksy-Wawrzyniak, Joanna Kozłowska, Przemysław Gagat, Agnieszka Grabowiecka

**Affiliations:** 1 Department of Microbiology, Faculty of Medicine, Wroclaw Medical University, Wroclaw, Poland; 2 Department of Bioorganic Chemistry, Wrocław University of Science and Technology, Wrocław, Poland; 3 Faculty of Medicine, Wroclaw Medical University, Wroclaw, Poland; 4 Department of Pharmaceutical Microbiology and Parasitology, Faculty of Pharmacy, Wroclaw Medical University, Wroclaw, Poland; 5 Department of Food Chemistry and Biocatalysis, Faculty of Biotechnology and Food Science, Wrocław University of Environmental and Life Sciences, Wrocław, Poland; 6 Faculty of Biotechnology, University of Wroclaw, Wroclaw, Poland

**Keywords:** Anti-biofilm activity, catheter-associated infections, flow conditions, gut-skin-microbiota axis, naringenin derivatives

## Abstract

The capacity for biofilm formation is a fundamental defense mechanism among antimicrobial-resistant pathogenic strains. In addition, its persistence may result in chronic colonization of host systems, and uncontrolled growth may lead to dangerous flow blockages, particularly in catheter-associated urinary tract infections. This warrants the search for novel anti-biofilm compounds to combat these pathogens. Naringenin is an example of such a structure, widely known for its multifunctional activity, targeting the synthesis of exopolysaccharide, the expression of biofilm-relevant genes, or efflux pump activity. In this study, we present a series of *O*-alkyl derivatives of naringenin and its oximes, that exhibited antimicrobial and biofilm-reducing activity against clinical strains of *Escherichia coli* and *Staphylococcus aureus*, which are often the cause of urinary tract infections. Most of the derivatives were highly active against the planktonic form of bacteria, the most potent of them being 7-*O*-methylnaringenin oxime, diminishing the growth of *E. coli* and *S. aureus* by 43.2% and 74.6%, respectively. The initial screening of the antibiofilm capabilities of the derivatives was performed in static conditions in a gravimetric method utilizing quartz tuning forks. While most of them were significantly less active than against the planktonic form, the oximes of 7-*O*-methylnaringenin and 7-*O*-isopropylnaringenin were found to impair the growth of biofilm in case of both strains. Therefore, the observed reduction in biofilm mass under static conditions may reflect not only antimicrobial activity but also biofilm-specific mechanisms, as indicated by the use of flow conditions with catheters simulating urine flux. These studies confirmed the activity of 7-*O*-methylnaringenin oxime, which reduced *E. coli* CCM 5712 population.

## 1 Introduction

Hospital-acquired infections pose a serious clinical challenge worldwide. Among these, catheter-associated urinary tract infections (CAUTIs) account for up to 40% of all cases (Hooton et al., 2010; Tambyah and Maki, 2015). According to the criteria of the Centers for Disease Control and Prevention (CDC) National Healthcare Safety Network (NHSN), a CAUTI is defined as a symptomatic urinary tract infection (SUTI) that occurs in a patient who has had a urinary catheter in place for at least two consecutive days before the onset of symptoms (Melzer and Welch, 2013a; Use et al., 2025). Moreover, indwelling urinary catheterization represents the most common factor in complicated urinary tract infections, often leading to secondary bloodstream infections (Warren, 1997; Melzer and Welch, 2013b; Tambyah and Maki, 2015; Trautner and Darouiche, 2015), and is associated with increased mortality (Ackermann and Patrick, 1996). The most severe clinical complication is urosepsis. Although its global prevalence is difficult to determine, published data suggest that it may account for as many as 10-30% of all causes of severe sepsis or septic shock reported each year worldwide (Wagenlehner et al., 2013). The mortality rate ranges from 14% to as high as 60%, depending on the study population and geographic region (Peach et al., 2016; Holmbom et al., 2022), and most often affects people over 65 years of age (Rosser et al., 1999; Kalra and Raizada, 2009).

Given the physiological overlap between intestinal colonization and urinary tract infections’ pathways, *E. coli* and *S. aureus* are among the most common causative agents of CAUTI. *E. coli* accounts for the vast majority (54.1% of 80.3%) of Gram-negative bacteria isolated from cases of urosepsis, whereas *S. aureus* predominates (13.1% of 19.7%) among Gram-positive isolates (Ackermann and Patrick, 1996). Easier trans-epithelial and hematogenous translocation of bacteria is linked with: i) disrupted intestinal barrier (“leaky gut”) (Turner, 2009; Camilleri et al., 2012; Brigida et al., 2024); ii) intestinal dysbiosis (Iqbal et al., 2024); iii) poor hygiene and sexual behaviors (Worby et al., 2022); iv) anatomical factors (Meštrović et al., 2021); and v) reduced immunity (Miller et al., 2024; Calin et al., 2025). This directly contributes to the migration of multidrug-resistant (MDR) strains, which, after translocating from the intestinal lumen, colonize periurogenital areas and subsequently enter the urinary tract through ascending infection and/or contaminated medical equipment (Brigida et al., 2024). Such processes also affect the gut-skin-microbiota axis (GSMA), which becomes enriched with intestinal MDR pathogens – a phenomenon of particular clinical relevance during the use of percutaneous catheters (Zhao et al., 2025).

**Figure 1:**
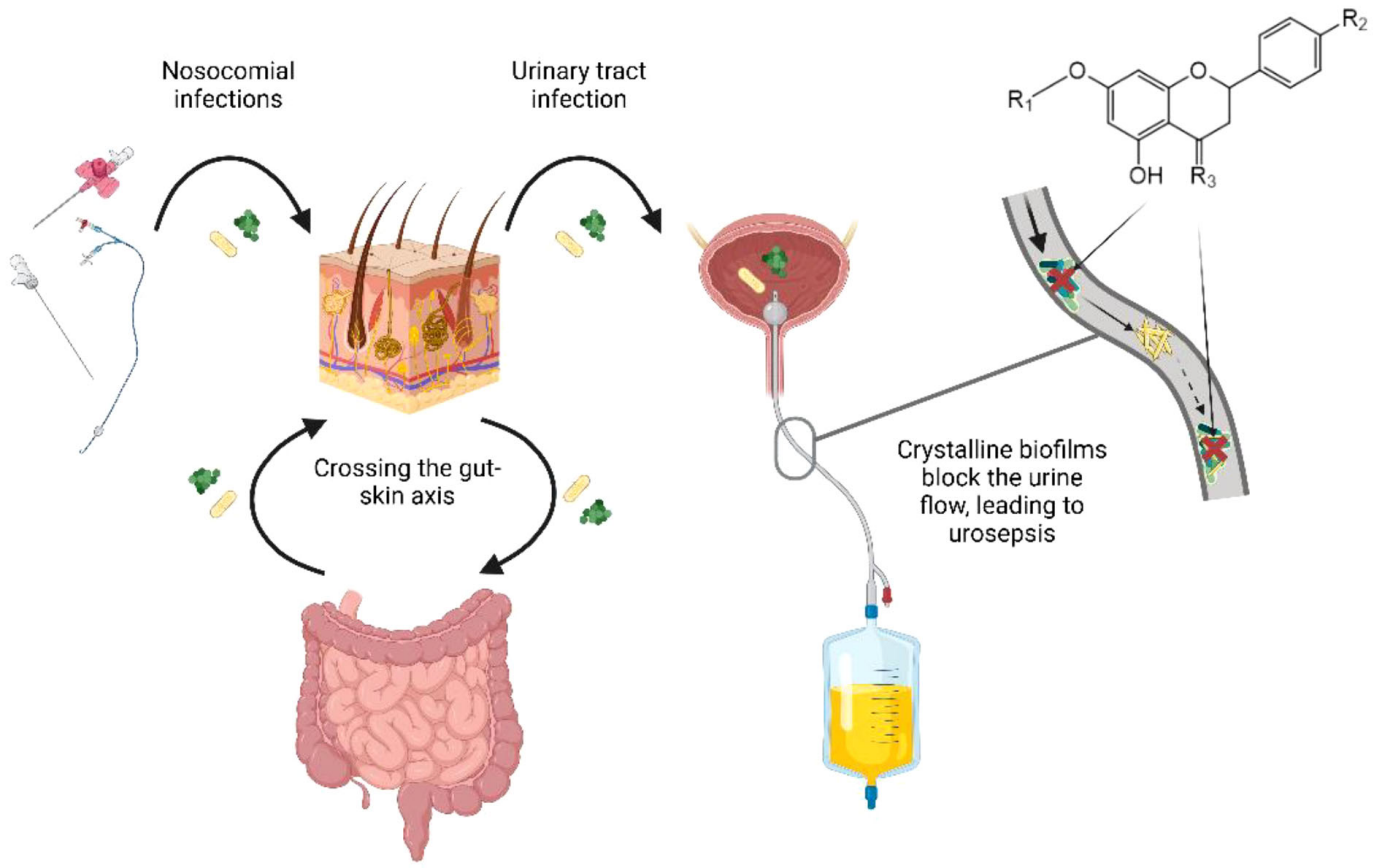
The role of the gut-skin-microbiota axis in catheter-associated urinary tract infections (CAUTIs).

The gastrointestinal tract serves as a natural reservoir for biofilm-forming bacteria – a three-dimensional structure composed of bacterial cells embedded in an extracellular matrix – capable of withstanding environmental stress, evading immune responses, and resisting antimicrobial agents. In addition, intestinal biofilms provide bacteria with a protective niche that facilitates: i) survival (Donlan and Costerton, 2002), ii) horizontal gene transfer (including resistance genes) (Metzger et al., 2022), and iii) potential translocation to surrounding areas, including the urinary tract (Brigida et al., 2024). Due to their virulence factors, these pathogens are capable of forming biofilms on implanted medical devices. Many uropathogenic bacteria originating from the intestinal reservoir produce urease, which contributes to the formation of ammonium-magnesium phosphate (struvite) and calcium phosphate crystals. These minerals become integrated into the extracellular polymeric substances (EPS) matrix of the biofilm, leading to the formation of crystalline biofilms. This additional structural component further stabilizes the three-dimensional microbial community and obstructs urinary flow, thereby promoting chronic urinary tract infections and CAUTIs (Sabir et al., 2017; Razi et al., 2024). This phenomenon is frequently observed in patients with long-term catheterization or intestinal dysbiosis. Therefore, preventing the transfer of these bacteria to other anatomical sites is an important strategy in reducing chronic and hospital-acquired infections associated with biofilm formation, which account for more than 60% of such cases (Hall-stoodley et al., 2004). This phenomenon contributes to the potential threat of ever-increasing antibiotic resistance, one of the greatest challenges of modern medicine. These correlations are illustrated in **Figure 1**.

**Figure 2:**
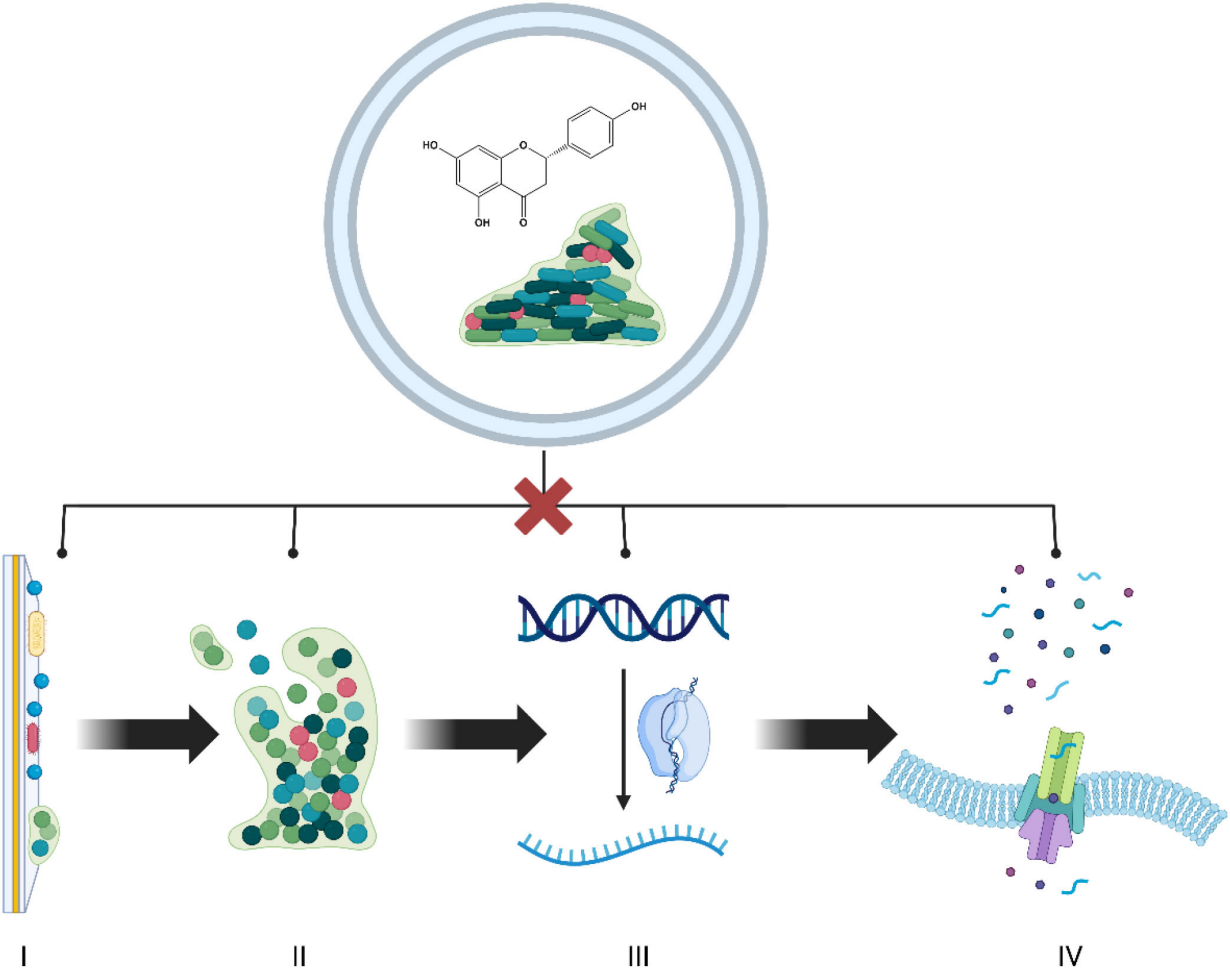
Anti-biofilm activity of naringenin. The figure shows the multifaceted inhibitory effects of NG on key stages of biofilm development. NG disrupts: microbial Surface adhesion, limiting initial attachment to abiotic and biotic surfaces (I); the production of exopolysaccharides (EPS), reducing the structural stability of the biofilm matrix (II); the expression of quorum-sensing-relevant genes, disrupting cell-to-cell bacterial communication (III), and efflux pump’s activity, increasing bacterial susceptibility to antimicrobial agents. In total, these mechanisms contribute to the impairment of biofilm formation and stability.

Naringenin (NG), a naturally occurring citrus flavonoid, exhibits a broad spectrum of beneficial biological activities, including anti-biofilm (Song et al., 2020), antioxidant (Ahmad et al., 2022), anti-inflammatory (Cai et al., 2023), and antimicrobial (Duda-Madej et al., 2020) effects. Recent studies indicate that this compound can modulate quorum sensing (QS) and biofilm formation in various bacterial species, including *E. coli* (Vikram et al., 2010) and *S. aureus* (Wen et al., 2021). The anti-biofilm activity of NG comprises multiple essential mechanisms and involves multidirectional modulation of processes strictly associated with biofilm development. NG disrupts biofilm formation directly by: i) interfering with cell-cell communication systems (e.g., inhibition of QS-regulated gene transcription such as *lasI* and *rhlI*) (Hernando-Amado et al., 2020), ii) reducing microbial adhesion capacity (Duda-Madej et al., 2023), iii) reducing its mass, increasing the effectiveness of antibiotic therapy (blocking NorA pumps in *S. aureus*) (Menezes Dantas et al., 2025), and iv) impairing biofilm surface and mutation (e.g., decreasing the production of exopolysaccharides responsible for structural stability) (Wen et al., 2021). Indirectly, it acts by: i) oxidative stress, and ii) reducing local inflammation, thereby hindering microbial persistence within the biofilm matrix (Duda-Madej et al., 2022; Cai et al., 2023). The anti-biofilm activity of NG is shown in **Figure 2**.

Recent scientific reports have demonstrated that chemical modification of NG – particularly *O*-alkylation and oxime formation – enhances its antimicrobial activity (Duda-Madej et al., 2020; Duda-Madej et al., 2023). Despite the relatively extensive literature on NG derivatives, their anti-biofilm potential has not yet been explicitly confirmed. In the present study, we evaluated a series of *O*-alkyl NG derivatives and their oximes for their ability to inhibit bacterial biofilm formation and planktonic growth under conditions mimicking the urinary tract environment. We selected clinically relevant species, *E. coli* and *S. aureus*, commonly implicated in extraintestinal CAUTI. The primary aim of this study was to assess how structural modifications of NG affect biomaterial colonization and anti-biofilm activity against gut-derived uropathogens under clinically significant conditions.

## 2 Materials and methods

### 2.1 Tested strains

The selection of bacterial strains was based on the epidemiology and pathogenesis of catheter-associated urinary tract infections. Most CAUTIs are caused by microorganisms from the patient’s own microbiota, in particular intestinal bacteria, i.e., *Escherichia coli* which colonize the gastrointestinal tract and enter the surface of the catheter through the periurethral area. Therefore, five fecal isolates exhibiting an extended-spectrum beta-lactamase (ESBL)-producing phenotype: B27, B29, B33, B41, and B55 were included in this study as clinically relevant representatives of potential uropathogens. In addition, the study also included pathogens colonizing the skin, and thus the site of catheter insertion, contributing to medical device-associated infections. Five isolates from wounds (*Staphylococcus aureus)* demonstrated a methicillin-resistant phenotype (MRSA): MR7, MR8, MR10, MR11, and MR24 were therefore considered to be appropriate clinical models of relevant strains with high potential for biofilm formation on medical surfaces. This approach adequately reflects the concept of the gut-skin-urinary tract axis, in which microorganisms from both the intestinal and skin reservoirs can contribute to catheter-related infections. The resistance profile of the tested strains is presented in **Table 1**. The studies included the following representative of the reference strains: *E. coli* CCM 5172 and *S. aureus* PCM 2054.

**Table 1:** Antimicrobial profiles determined by Phoenix system.

Strain	Antimicrobial resistance profile	Resistance phenotype
*S. aureus isolates*		
MR7	E^R^, CL^R^, CIP^R^, FOX^R^,G^S^, T^S^	cMLSB, MRSA
MR8	E^R^, CL^R^, CIP^R^, FOX^R^, G^S^, T^S^	cMLSB, MRSA
MR10	E^R^, FOX^R^, CL^S^, CIP^S^, G^S^, T^S^	iMLSB, MRSA
MR11	CIP^R^, FOX^R^, E^S^, CL^S^, G^S^, T^S^	MRSA
MR24	E^R^, CL^R^, CIP^R^, FOX^R^, G^S^, T^S^	cMLSB, MRSA
*E. coli isolates*		
B27	AN^R^,TOB^R^,G^R^, AMC^R^, SAM^R^, CAZ^R^, CTX^R^, CXM^R^, ATM^R^, TZP^R^, FEP^R^, SXT^R^, CIP^S^, IPM^S^, MEM^S^, DOR^S^, ETP^S^	ESBL
B29	G^R^, AMC^R^, SAM^R^, CAZ^R^, CTX^R^, CXM^R^, ATM^R^, TZP^R^, FEP^R^, SXT^R^, AN^SIE^, TOB^SIE^, CIP^S^, IPM^S^, MEM^S^, DOR^S^, ETP^S^	ESBL
B33	TOB^R^, G^R^, AMC^R^, SAM^R^, CIP^R^, CAZ^R^, CTX^R^, CXM^R^, ATM^R^, TZPR, FEP^R^, SXT^R^, AN^SIE^, IPM^S^, MEM^S^, DOR^S^, ETP^S^	ESBL
B41	AMC^R^, SAM^R^, CAZ^R^, CXM^R^, ATM^R^, TZP^R^, SXT^R^, AN^S^, TOB^S^, G^S^, CIP^S^, CTX^S^, FEP^S^, IPM^S^, MEM^S^, DOR^S^, ETP^S^	ESBL
B55	TOB^R^, G^R^, AMC^R^, SAM^R^, CIP^R^, CAZ^R^, CTX^R^, CXM^R^, ATM^R^, TZP^R^, FEP^R^, SXT^R^, AN^SIE^, IPM^S^, MEM^S^, DOR^S^, ETP^S^	ESBL

AMC, amoxicillin+clavualanic acid; AN, amikacin; ATM, aztreonam; CAZ, cefotaxime; CIP, ciprofloxacin; CL, clindamycin; cMLSB, constitutive resistance to macrolide, lincosamide and streptogramin B antibiotics; CTX, cefotaxime; CXM, cefuroxime; DOR, doripenem; E, erythromycin; ESBL, extended spectrum beta lactamase; ETP, ertapenem; FEP: cefepime; FOX, cefoxitin; G, gentamicin; iMLSB, inducible resistance to macrolide, lincosamide and streptogramin B antibiotics; IPM, imipenem; MEM, meropenem; MRSA, methicillin-resistant *Staphylococcus aureus*; ^R^ resistant; ^S^ susceptible; SAM, ampicillin+sulbactam; ^SIE^ sensitive increased exposure; SXT, trimethoprim-sulfamethoxazole; T, tetracycline; TOB, tobramycin; TZP, piperacillin+tazobactam.

All bacterial strains used in the present study originated from the strain collections of the Department of Pharmaceutical Microbiology, Wroclaw, Medical University (resistant clinical isolates), and the Department of Bioorganic Chemistry, Wroclaw University of Science and Technology (reference strains).

#### 2.1.1 Revival of bacterial strains

The bacterial strains *S. aureus* and *E. coli* were reactivated from preserved museum stocks prior to experimental use. For cryopreserved stocks stored at -80 °C in 20% glycerol, they were rapidly thawed at 37 °C and added to the respective medium, tryptic soy broth (TSB) for S. aureus and Luria-Bertani broth (LB) for *E. coli*, gently mixed, and incubated for 24 h at 37 °C. After that, 100-200 µL of culture was streaked onto blood agar (Columbia agar, Oxoid) and MacConkey agar (Oxoid) plates for *S. aureus* and *E. coli*, respectively. The inoculated plates were incubated at 37 °C for 18–24 h under aerobic conditions.

### 2.2 Naringenin derivatives tested

Synthesis of *O*-alkyl derivatives of NG (1a, 2a, 4a, 10a, 12a, and 14a) and their oximes (1b, 2b, 4b, 10b, 12b, and 14b) was described in our previous works (Kozłowska et al., 2017; Kozłowska et al., 2019). Briefly, to NG dissolved in anhydrous acetone, anhydrous potassium carbonate and appropriate alkyl iodide were added. The reaction was conducted until the NG was completely reacted and the desired products were obtained. Then, the solvent was removed, a saturated solution of sodium chloride was added and extraction with an organic solvent was performed. Collected extracts were dried, concentrated on vacuum evaporator and purified by liquid column chromatography. Oximes of *O*-alkyl derivatives of NG were obtained by reaction with hydroxylamine hydrochloride and anhydrous sodium acetate in anhydrous ethanol. After complete conversion of *O*-alkyl derivative of NG, the reaction mixture was poured into ice water, the precipitated crystals were collected and purified by liquid column chromatography. The *O*-alkyl derivatives of naringenin and their oximes for testing were prepared as a fresh stock solution 20 mg mL^-1^in DMSO. The prepared concentration allowed for complete dissolution of the tested compounds and sampling of a small amount for further testing in order to avoid turbidity and maintain their stability.

### 2.3 Gravimetric biofilm evaluation using quartz tuning forks

The system and software used in the gravimetric measurements of biofilm formation were kindly provided by the Department of Nanometrology, Faculty of Electronics, Photonics and Microsystems, Wrocław University of Science and Technology (Piasecki et al., 2015).

Mueller-Hinton broth (MHB; Oxoid; 100 µL) with or without NG derivatives at concentrations of 100 µg mL^-1^ was used as the growth medium in a 96-well plate. The selected concentration represents the highest experimentally feasible dose under aqueous conditions without visible precipitation of the tested compounds. The concentration of the compounds for this screening was selected on the basis of the initial studies of their activity against pathogenic strains, while taking into consideration their limited solubility in aqueous environment. The final number of microbial cells per well was 5×10^4^ CFU/mL. The conductance of quartz tuning forks (QTFs) was measured using QTF computer software. The tuning forks were considered mechanically undamaged if their conductance was within the range of 6-12 µS. They were sterilized in ethanol for 20 minutes, left for drying, and checked again for possible mechanical damage while kept in a sterile environment. Their resonant frequency was also measured. Afterwards, they were dipped in prepared cell suspensions. Growth control without the forks was performed to confirm that the metal in the tuning forks does not have an influence on the growth of the studied microorganisms. After three days of incubation, QTFs were removed from the wells of the microplate, dried, and their resonating frequency was measured. A drop in frequency (in comparison to the values before the assay) was directly proportional to the mass of biofilm burdening the forks according to the following formula: f [mHz] = 18.517*m [ng]. Optical density OD_650_ measurement of the cell cultures and MTT assay were performed to assess the influence of NG derivatives on the growth and viability of planktonic form. The latter was conducted by adding the 3-(4,5-dimethylthiazol-2-yl)-2,5-diphenyltetrazolium bromide to the concentration of 0.5 mg mL^-1^ and solubilization of the acquired crystals with 1.5% HCl in isopropanol in the 1:1 volume ratio to the cell cultures.

### 2.4 Antimicrobial activity of naringenin and its derivatives

The antimicrobial properties were tested *in vitro* using the minimum inhibitory concentration (MIC) microdilution method, as recommended by the European Committee on Antimicrobial Susceptibility Testing (EUCAST) (The European Committee on Antimicrobial Susceptibility Testing, 2020). The assays were performed in MHB medium using 96-well microdilution plates. *O*-alkyl derivatives of NG and their oximes were tested at concentrations ranging from 512 to 1 µg mL^-1^ in geometric progression. The concentration of test strains in each test well was 5x10^5^ CFU/mL. Optical density was read at 600 nm after 18 h incubation at 37 °C in a titration plate reader (ASYS UVM340, BIOCHROM Ltd., Cambridge, UK). The test was set in triplicate, each in 3 independent replicates. In addition, controls were set for each assay: background absorbances of sterile MHB and compound’s solutions in sterile MHB and bacterial strain growth in MHB and in DMSO at a concentration of 0.01-5.12%. The concentration at which the compound caused 90% or 50% growth inhibition of the bacterial strain (relative to the growth control) was considered the MIC_90_ and MIC_50_ values, respectively.

To determine the minimum bactericidal concentration (MBC), 10 µL aliquots of broth from the well showing MIC and the adjacent wells were seeded onto tryptose-soy agar (TSA). The plates were incubated for 24 h at 37°C, after which the growth of the tested bacterial strains was visually assessed by counting the cultured bacterial colonies. The assay was performed three times for each well.

### 2.5 Biofilm evaluation under flow-through conditions

**Figure 3:**
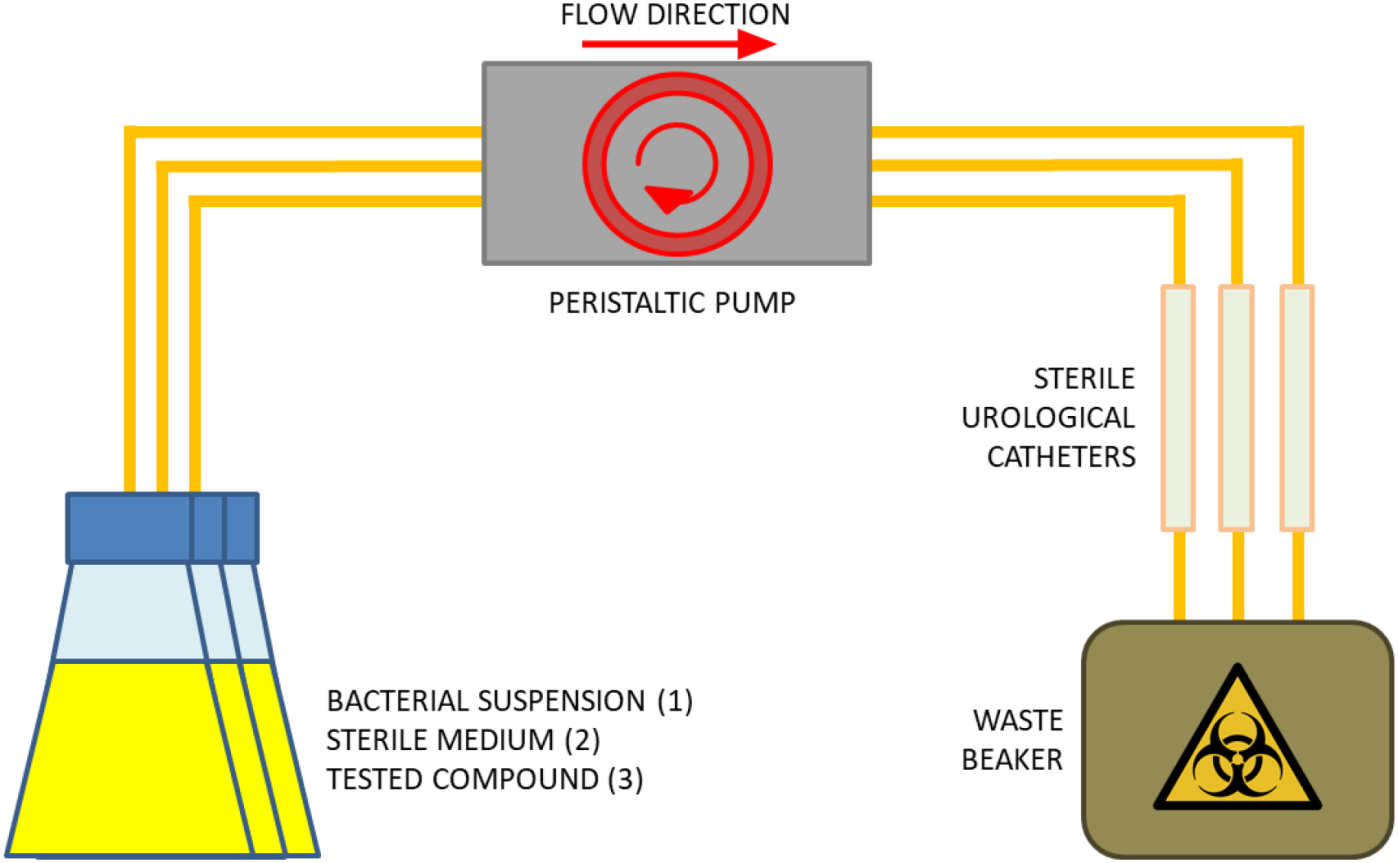
Schematic representation of flow-based biofilm formation system. The setup consists of a peristaltic pump that drives continuous medium flow from a reservoir flask through three parallel catheter segments. Following exposure to bacterial suspensions (1), catheter fragments are perfused with fresh medium to allow biofilm development (2), after which tested compounds are introduced under identical flow conditions – 0.5 mL/min (3). Effluent is collected in a biohazard container. The system mimics physiological flow dynamics relevant to catheter-associated biofilm formation in the urinary tract.

The system for evaluating the anti-biofilm activity of the tested compounds under flow conditions consisted of a series of sterile polyvinyl chloride Nelaton-type catheters (GalMed, Bydgoszcz, Poland) connected to a peristaltic pump (Ismatec, Cole-Parmer GmbH, Wertheim, Germany) based on a previous study (Oleksy-Wawrzyniak et al., 2022). Experiments were conducted under conditions stimulating the urinary tract environment, with a flow rate through the catheters of 0.5 mL/min, corresponding to the rate of final urine formation. The entire system was placed in an incubator maintained at a constant temperature of 37 °C to simulate the urinary tract environment. The structural layout of the system is shown in **Figure 3**.

Bacterial suspensions were prepared in MHB medium at a density of 1.0 McFarland (MF), determined densitometrically (Densimat, BioMérieux) These suspensions were then passed through a set of three urological catheters for two hours under defined flow conditions. Subsequently, the terminal catheter fragments exposed to bacterial suspensions were replaced with sterile ones, and transferred individually into a bottle containing fresh MHB medium. Continuous flow was maintained for 24 hours at 37°C to allow biofilm formation. Following biofilm development, solutions of the tested compounds at 100 µg mL^-1^ (in MHB) were perfused through the system for three hours. Afterwards, the catheter fragments were removed, aseptically cut, and subjected to quantitative biofilm evaluation. Positive (the tested strain itself) and negative (pure MHB medium) controls were included in each experiment. All procedures were performed in three independent replicates.

### 2.6 Quantitative assessment of biofilm under flow conditions

The biomaterial was cut aseptically into 1 cm long pieces. The three middle fragments were placed in tubes containing 2 ml of 0.5% saponin (Merck, Darmstadt, Germany), and the tubes were vigorously shaken on a vibrating shaker (Microspin FV-2400, BioSan, Józefów, Poland) for 1 minute to separate the bacterial cells adhering to the surface of the biomaterial. A series of six consecutive dilutions in sterile NaCl solution (Stanlab, Lublin, Poland) was prepared and quantitatively seeded onto dedicated media, MacConkey Agar (Becton Dickinson, Warsaw, Poland) for Gram-negative bacteria or Columbia Agar (Becton Dickinson, Warsaw, Poland) for Gram-positive bacteria. The plates were then incubated at 37 °C for 24 hours. After incubation, the number of bacterial colonies grown on the medium was counted. The test was performed in three replicates for each flow experiment, resulting in a total of nine technical replicates for each strain tested. Quantitative calculations were performed against positive controls, i.e., the pure test strain (100%), and a negative control representing pure MHB medium.

### 2.7 Statistical analysis

Values of raw plankton growth, planktonic cell viability, biofilm formation under static conditions, and biofilm formation under flow conditions were normalized to the mean DMSO control (X̄_DMSO_) within each bacteria × set (where applicable). Percent inhibition of plankton growth and biofilm formation (static and flow conditions) was calculated as 100 × (1 − X_i_/X̄_DMSO_), where X_i_ denotes an individual raw measurement for a given compound; results are presented as mean ± standard deviation. Planktonic cell viability was calculated as 100 × (X_i_/X̄_DMSO_) and is likewise presented as mean ± standard deviation. Statistical significance of inhibition of plankton growth, bacterial viability, and biofilm formation was assessed using one-sided Wilcoxon rank-sum tests, comparing each compound to the DMSO control. False-discovery rate correction was performed using the Benjamini–Hochberg procedure independently for each bacteria × set (where applicable). All statistical analyses and data visualizations were performed in R (version 4.5.1) using the *dplyr*, *ggplot2*, and *rstatix* packages.

## 3 Results

### 3.1 Antimicrobial and biofilm-protective activity under static conditions

A series of *O*-alkyl derivatives of NG (1a, 2a, 4a, 10a, 12a, and 14a), their oximes (1b, 2b, 4b, 10b, 12b, 14b), and NG as a standard have been verified for their potency to prevent bacterial biofilm formation. These compounds were previously chemically characterized, their purity and stability in solvent were assessed (based on NMR spectra) (Kozłowska and Duda-Madej, 2023), and their antiproliferative activity against fibroblast, 3T3-L1, and intestinal HT-29 cell lines was determined at various times for the purposes of these tests (Duda-Madej et al., 2023; Kozłowska and Duda-Madej, 2023). This study focused on their influence upon biofilm formation in *E. coli* CCM5172 and *S. aureus* PCM 2054 strains, assessed in a stationary model, and aimed to select compounds with the most substantial anti-biofilm potential. The characteristics of the compounds studied are presented in **Table 2**.

**Table 2:** Planktonic growth, metabolic viability (MTT), and biofilm formation (QTF) in *E. coli* CCM5172 and *S. aureus* PCM 2054 strains under experimental conditions.

Entry	Structure		Planktonic growth [%]	Viability (MTT) [%]	Biofilm formation (QTF) [%]
1a	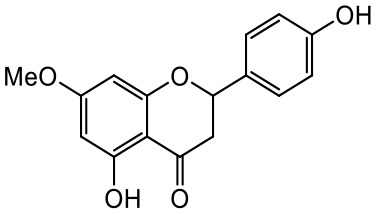 (1) 7-*O*-methylnaringenin	*E. coli*	81.5 ± 7.8	100	94.1 ± 5.2
		*S. aureus*	71.8 ± 9.3	80.4 ± 4.3	100
1b	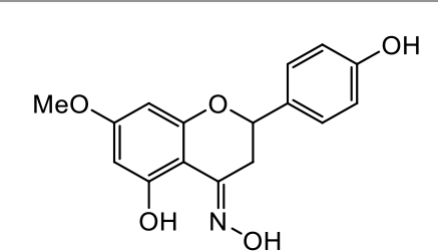 (2) 7-*O*-methylnaringenin oxime	*E. coli*	56.8 ± 2.7	79.7 ± 3.4	77.8 ± 3.7
		*S. aureus*	25.4 ± 0.8	21.7 ± 2.8	63.7 ± 5.2
2a	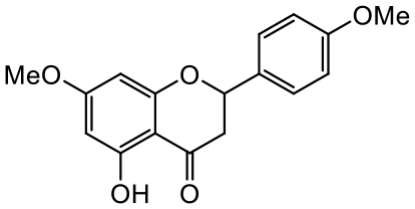 (3) 7,4’-di-*O*-methylnaringenin	*E. coli*	88.4 ± 4.3	97.4 ± 8.1	100
		*S. aureus*	100	100	100
2b	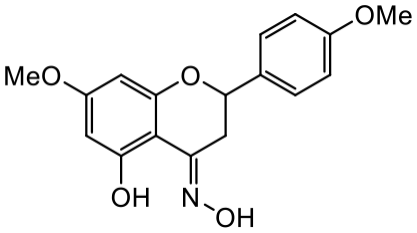 (4) 7,4’-di-*O*-methylnaringenin oxime	*E. coli*	92.9 ± 7.7	99.4 ± 8.3	100
		*S. aureus*	100	78.6 ± 8.2	100
4a	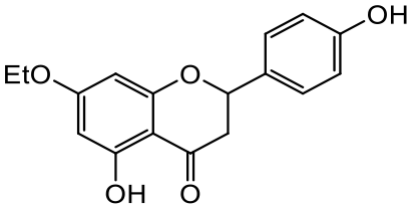 (5) 7-*O*-ethylnaringenin	*E. coli*	84.0 ± 5.3	100	100
		*S. aureus*	65.8 ± 2.0	63.4 ± 0.7	100
4b	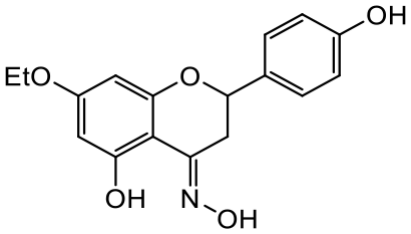 (6) 7-*O*-ethylnaringenin oxime	*E. coli*	69.8 ± 7.3	98.2 ± 2.0	70.4 ± 6.0
		*S. aureus*	31.8 ± 1.5	36.7 ± 1.8	100
10a	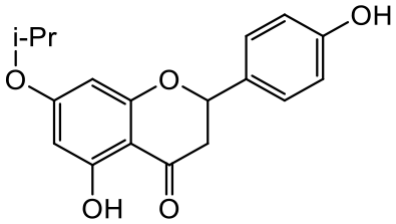 (7) 7-*O*-isopropylnaringenin	*E. coli*	98.6 ± 5.1	87.2 ± 4.9	93.4 ± 4.7
		*S. aureus*	44.3 ± 4.0	34.1 ± 1.7	54.2 ± 5.2
10b	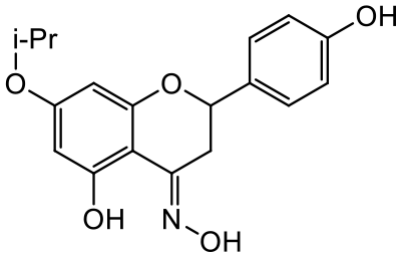 (8) 7-*O*-isopropylnaringenin oxime	*E. coli*	62.3 ± 4.3	81.6 ± 5.1	96.2 ± 6.8
		*S. aureus*	31.0 ± 0.4	23.2 ± 2.5	40.2 ± 4.1
12a	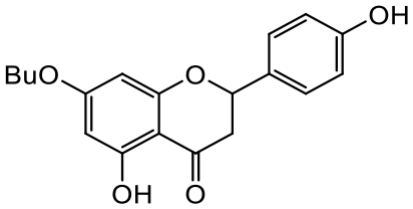 (9) 7-*O*-butylnaringenin	*E. coli*	72.5 ± 0.9	100	100
		*S. aureus*	34.5 ± 2.1	32.1± 0.4	100
12b	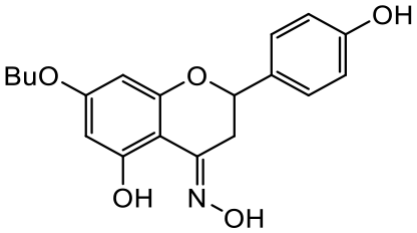 (10) 7-*O*-butylnaringenin oxime	*E. coli*	72.4 ± 5.2	96.2 ± 4.2	75.9 ± 4.6
		*S. aureus*	29.3 ± 0.4	45.2 ± 3.7	100
14a	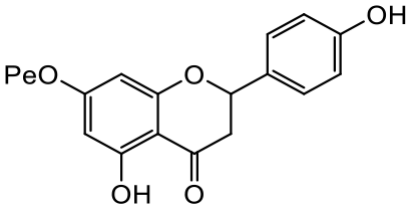 (11) 7-*O*-pentylnaringenin	*E. coli*	76.5 ± 6.2	100	72.5± 6.6
		*S. aureus*	46.8 ± 3.3	34.3 ± 0.3	100
14b	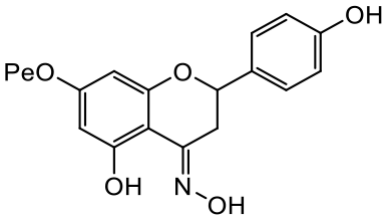 (12) 7-*O*-pentylnaringenin oxime	*E. coli*	71.8 ± 3.7	91.4 ± 4.0	93.1 ± 4.3
		*S. aureus*	27.3 ± 1.7	34.6± 0.8	100
NG	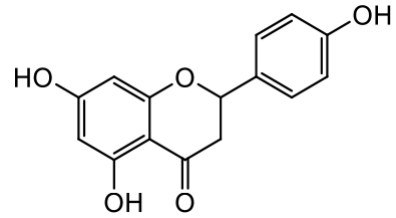 (13) naringenin	*E. coli*	70.7 ± 1.7	91.2 ± 6.9	64.5± 5.1
		*S. aureus*	72.1 ± 5.2	91.0 ± 3.8	89.4 ± 9.3

**Figure 4:**
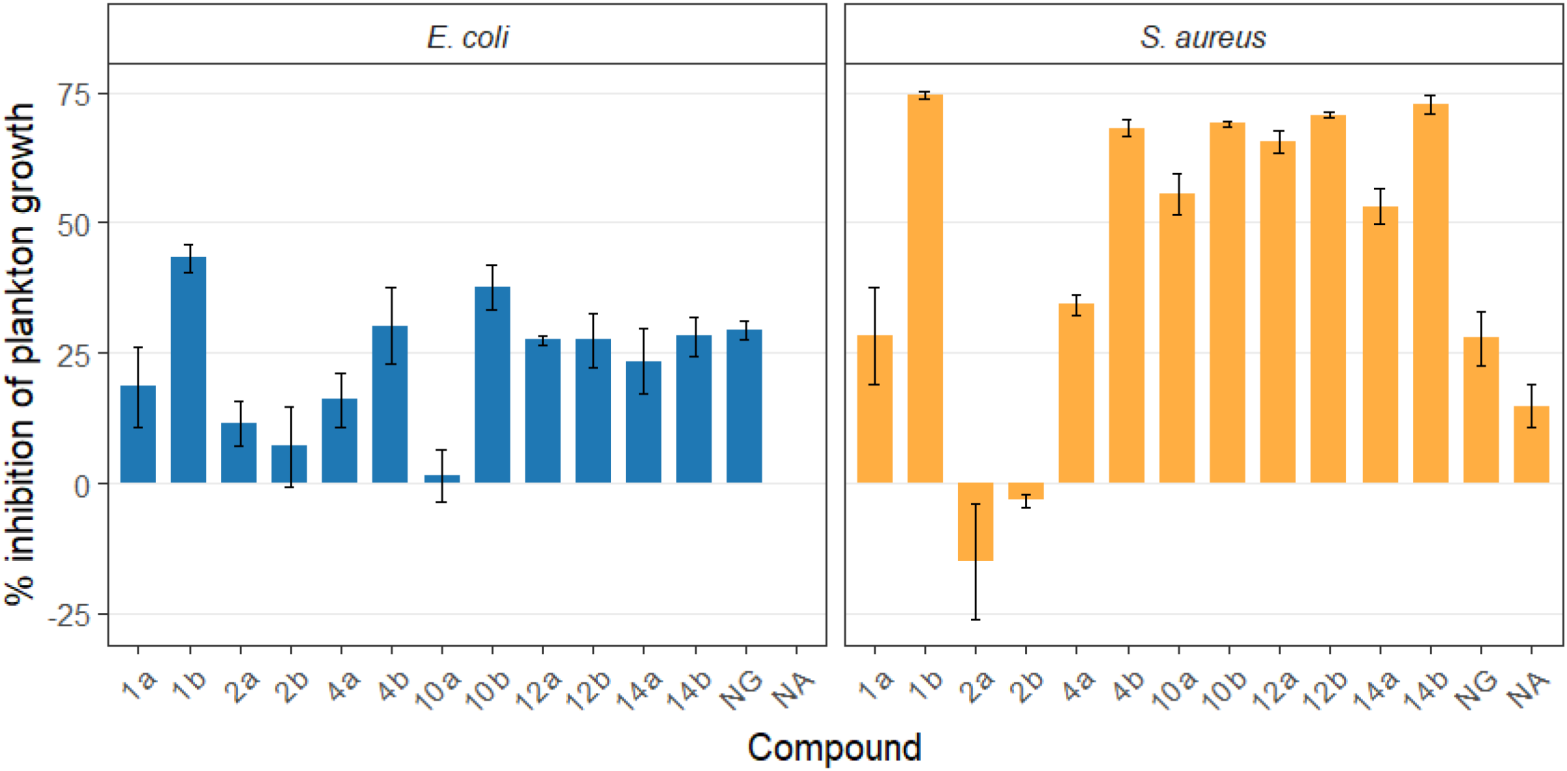
Percent inhibition of planktonic growth relative to the DMSO control for *E. coli* and *S. aureus*. Bars represent mean inhibition across replicates, and error bars denote standard deviation (SD). Positive values indicate reduced planktonic growth relative to DMSO, whereas negative values reflect no inhibition or slight growth enhancement; 1a, 2a, 4a, 10a, 12a, 14a - *O*-alkyl derivatives of naringenin; 1b, 2b, 4b, 10b, 12b, 14b - oximes of *O*-alkyl derivatives of naringenin; NG – naringenin; NA – not applicable (no compound added).

**Figure 5:**
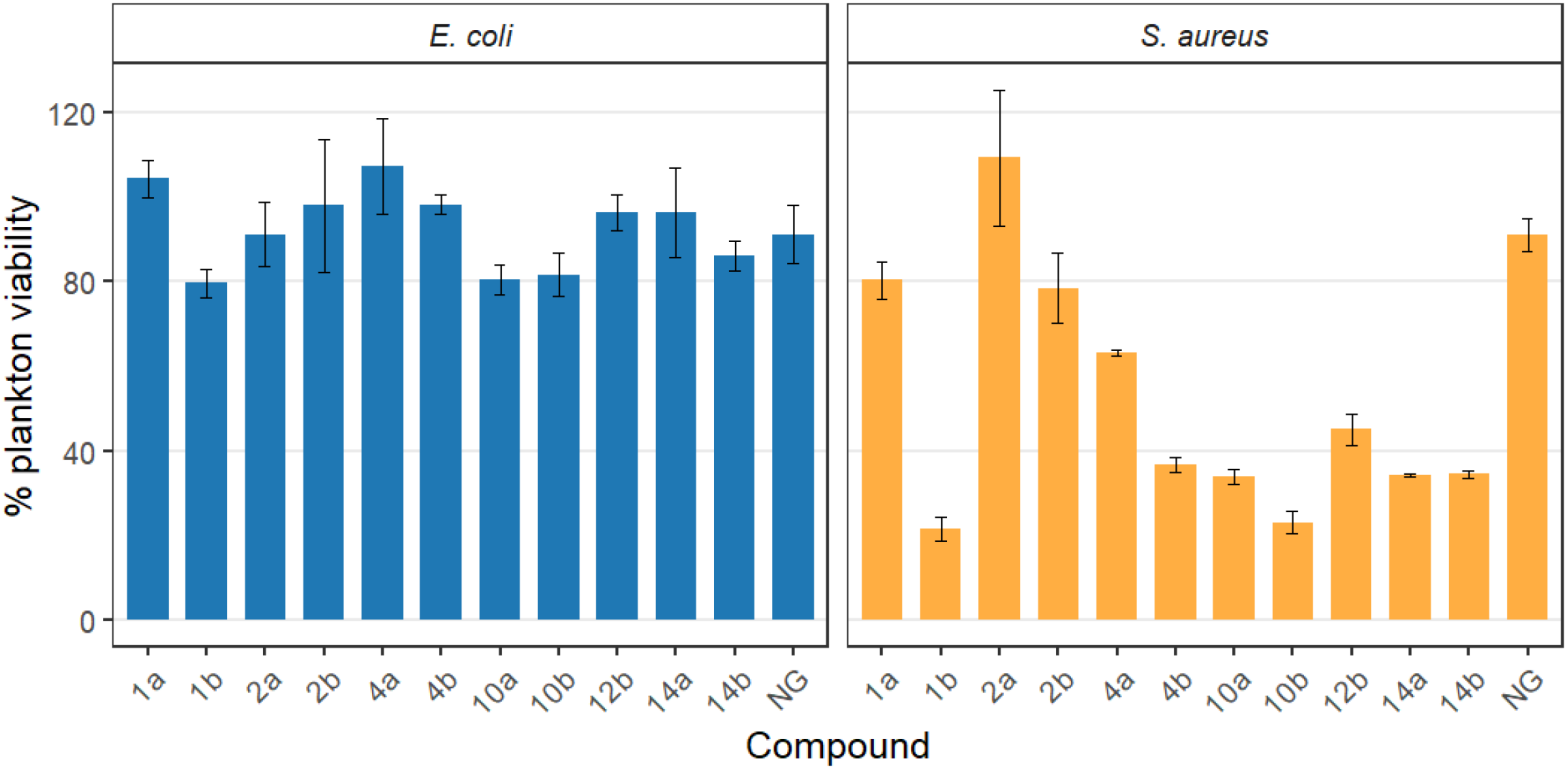
Planktonic cell viability of *E. coli* and *S. aureus* treated with the tested compounds. Viability was measured using the MTT assay and normalized to the DMSO control (100%). Bars show mean ± SD. *E. coli* viability remained largely unaffected, whereas several compounds substantially reduced *S. aureus* viability; 1a, 2a, 4a, 10a, 12a, 14a - *O*-alkyl derivatives of naringenin; 1b, 2b, 4b, 10b, 12b, 14b - oximes of *O*-alkyl derivatives of naringenin; NG - naringenin.

Preliminary screening experiments were performed with reference strains *S. aureus* PCM2054 and *E. coli* CCM 5172 cultured in the presence of 100 µg mL^-1^ of the tested compounds, which was five times below MIC_90_ against *E. coli* CCM 5172 for the most efficient oxime structures. The intensity of planktonic growth was evaluated with optical density measurements and the result of the MTT reduction assay. The amount of biofilm was assayed gravimetrically using a device based on QTF frequency measurements (Piasecki et al., 2015). The planktonic growth response to the tested compounds was notably more pronounced in *S. aureus* PCM2054 than in *E. coli* CCM 5172 cultures (**Supplementary Tables 1**, **S2**; **Figure 4**). In most of the compounds tested, the reduction in growth was more than twice as strong in the Gram-positive strain than in the Gram-negative strain, except for compounds 2a and 2b, which had negligible effects on both microorganisms tested. Overall, the oxime form of NG derivatives exhibited stronger antimicrobial activity. However, these observations were not reflected in the viability estimated by the MTT assay (**Figure 5**; **Supplementary Table 3**). Such discrepancies may occur when the studied antimicrobial agent interferes with the outer membrane of a Gram-negative microorganism, thereby increasing substrate influx and enhancing MTT formazan production (Grela et al., 2015). Unlike most of the compounds tested, the decrease in density in *E. coli* cultures caused by compounds 1b and 10b (by 43.2% and 37.7%, respectively, **Tables 2**, **S1**) was confirmed in simultaneous MTT assay estimates, in which the viability of bacterial cells ranged around 80% (**Supplementary Table 4**). This was the highest decrease in viability against this strain among the entire group of tested compounds. Notably, it was twice that of the reference compound used in the study, NG, for which a decrease in viability of approximately 9% was observed. Furthermore, these oximes (1b and 10b) exhibited significantly stronger activity against *S. aureus*, making these derivatives the most active of all the compounds tested against this strain. The growth of *S. aureus* in their presence was inhibited by 74.6% and 69% for 1b and 10b, respectively (**Supplementary Table 1**), and viability decreased below 25% (**Supplementary Table 4**). This result differs significantly from that obtained for NG, for which a more than 2.5-fold lower reduction in plankton (27.9%) was observed, while the reduction in viability was also 3-fold lower (9%) (**Supplementary Tables 1**, **S4**).

**Figure 6:**
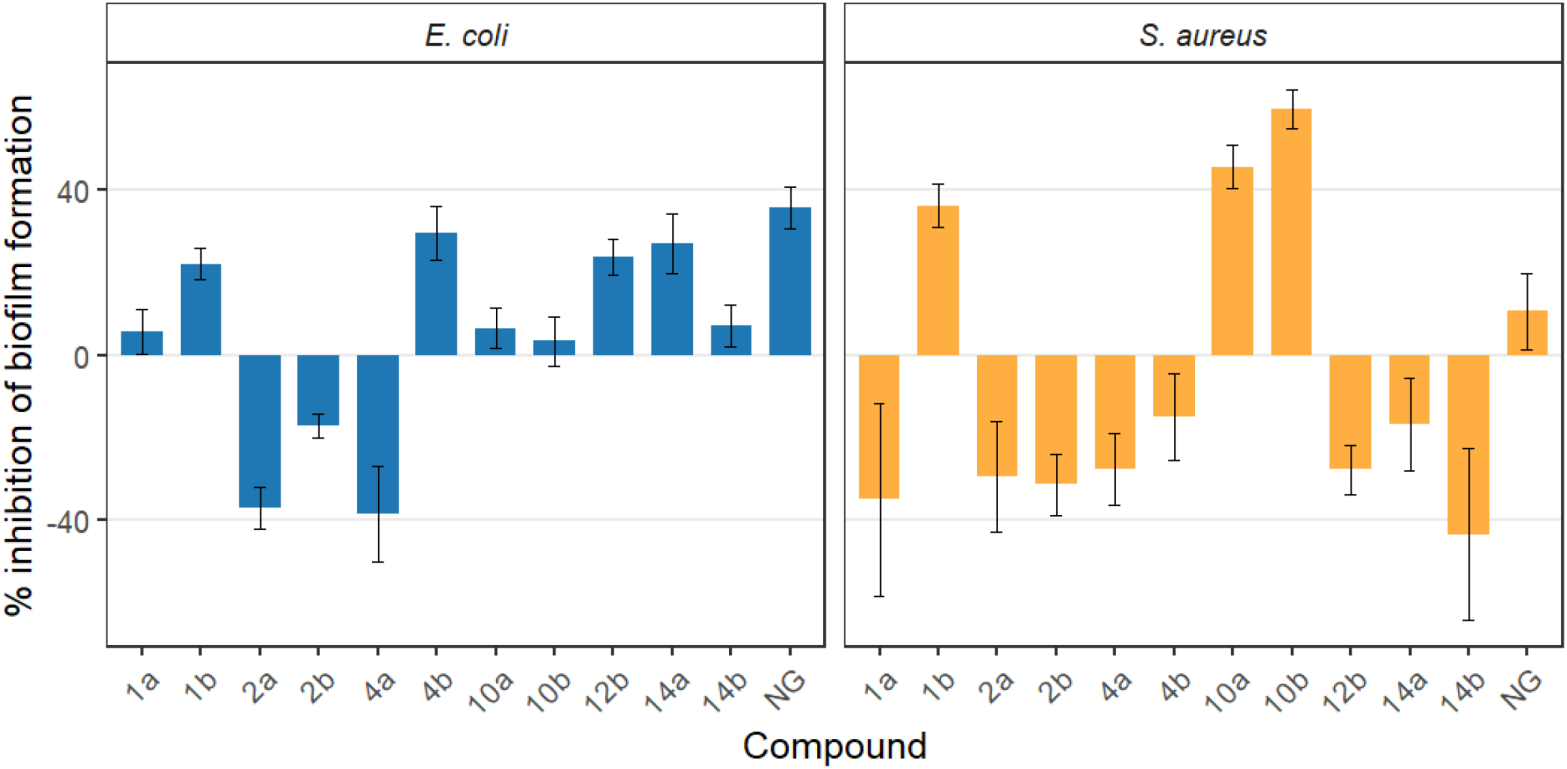
Percent inhibition of biofilm formation relative to the DMSO control for *E. coli* and *S. aureus*. Bars represent mean inhibition across replicates, and error bars denote standard deviation (SD). Positive values indicate reduced biofilm growth relative to DMSO, whereas negative values reflect no inhibition or growth enhancement; 1a, 2a, 4a, 10a, 12a, 14a - *O*-alkyl derivatives of naringenin; 1b, 2b, 4b, 10b, 12b, 14b - oximes of *O*-alkyl derivatives of naringenin; NG - naringenin.

The mass of bacterial biofilm built up on the surfaces of quartz tuning forks in the presence of compounds at a single concentration of 100 µg mL^-1^ was calculated after 3 days of incubation. It is to be noted that in this part of the study the reference strains were grown in the presence of the tested compounds from the moment of inoculation. Thus, the observed decrease in biomass attached to quartz tuning forks could result solely from antibacterial effect exerted on planktonic cells’ suspensions, reduced ability of cells to adhere, form and maintain the biofilm structure, or both. Under the test conditions employed, it was not possible to quantify the contribution of these mechanisms to the end-point result of biofilm dry mass decrease. The biofilm formation on tuning-fork surfaces was markedly reduced for both tested strains only by compounds 1b and 10b, which yielded values of 77.8% vs 63.7% for the former and 96.6% vs 40.2% for the latter, for *E. coli* and *S. aureus*, respectively (**Figure 6**; **Supplementary Tables 5**, **S6**). The other compounds showed no meaningful activity and affected biofilm development only slightly and solely with respect to one strain. Nevertheless, no *E. coli* biofilm buildup above the untreated control was observed despite the sub-inhibitory concentrations of the two antimicrobials, which suggested that the biofilm formation ability of the strain could be disturbed. This was confirmed further in the flow-through experiment, the results of which demonstrated activity of the compounds against mature biofilm established under optimal conditions. NG inhibited biofilm formation by *E. coli* at 64.2%, a level comparable to compound 1b, whereas its effect on *S. aureus* (89.4%) was substantially weaker than that observed for 1b (63.7%) and 10b (40.2%) (**Supplementary Tables 5**, **S6**).

Based on the above results, compounds 1b and 10b, as well as NG, which is the standard, were selected for further testing on clinically relevant strains. Notably, in the case of compounds 1b and 10b, the reduction in biofilm formation capacity occurred simultaneously with a significant decrease in plankton growth, suggesting that the observed effect may be partly due not only to a biofilm-specific mechanism, but also to the antimicrobial activity of these compounds.

### 3.2 Minimal inhibitory and bactericidal concentrations assay

To verify the difference in antimicrobial activity between the compounds selected for further testing and their effect on plankton, their activity against reference strains was compared with that against strains exhibiting MRSA and ESBL resistance profiles. In this study, NG and its two derivatives, 1b and 10b, showed negligible activity against all *E. coli* strains, with MIC_90_ and MBC values exceeding 512 µg mL^-1^ (**Table 3**).

**Table 3:** MIC and MBC values of naringenin and its derivatives against tested bacterial strains [µg mL^-1^].

Bacterial strain	MIC_50_/MIC_90_/MBC values for NG and its derivatives [µg mL^-1^]		
	1b	10b	NG
*S. aureus* MR7	1/16/64	1/8/64	1/128/>512
*S. aureus* MR8	1/16/64	1/16/64	4/64/>512
*S. aureus* MR10	1/16/128	1/16/64	1/128/>512
*S. aureus* MR11	1/16/128	1/16/64	1/64/>512
*S. aureus* MR24	1/32/128	1/32/64	1/64/>512
*S. aureus* PCM 2054	1/8/128	1/16/128	1/128/>512
*E. coli* B27	64/>512/>512	>512/>512/>512	512/>512/>512
*E. coli* B29	64/>512/>512	64/>512/>512	512/>512/>512
*E. coli* B33	>512/>512/>512	>512/>512/>512	512/>512/>512
*E. coli* B41	128/>512/>512	128/>512/>512	>512/>512/>512
*E. coli* B55	128/>512/>512	>512/>512/>512	512/>512/>512
*E. coli* CCM 5172	64/>512/>512	64/>512/>512	512/>512/>512

In contrast, both NG derivatives showed promising results against Gram-positive bacteria (6 strains of *S. aureus*). Compound 10b was particularly active, showing the highest activity against strain MR7 (MIC_90_/MBC = 8/64 µg mL^-1^), moderate activity against strains MR8, MR10, and MR11, similar to PCM2054 (MIC_90_/MBC = 16/64 µg mL^-1^), and the lowest against the MR24 strain. Meanwhile, derivative 1b showed the highest efficacy only against the reference strain (MIC_90_/MBC = 8/128 µg mL^-1^). In turn, against MRSA strains, it showed a MIC_90_/MBC value of 16/64 µg mL^-1^ for MR7 and MR8, 16/128 µg mL^-1^ for MR10 and MR11, and 32/128 µg mL^-1^ for MR24. Overall, compound 10b showed better antibacterial activity compared to compound 1b. NG, on the other hand, showed lower activity than the tested derivatives against all tested *Staphylococcus* strains, with MIC_90_ values of 64–128 µg mL^-1^ and MBC >512 µg mL^-1^ (**Table 3**).

### 3.3 Anti-biofilm activity under flow conditions

**Figure 7:**
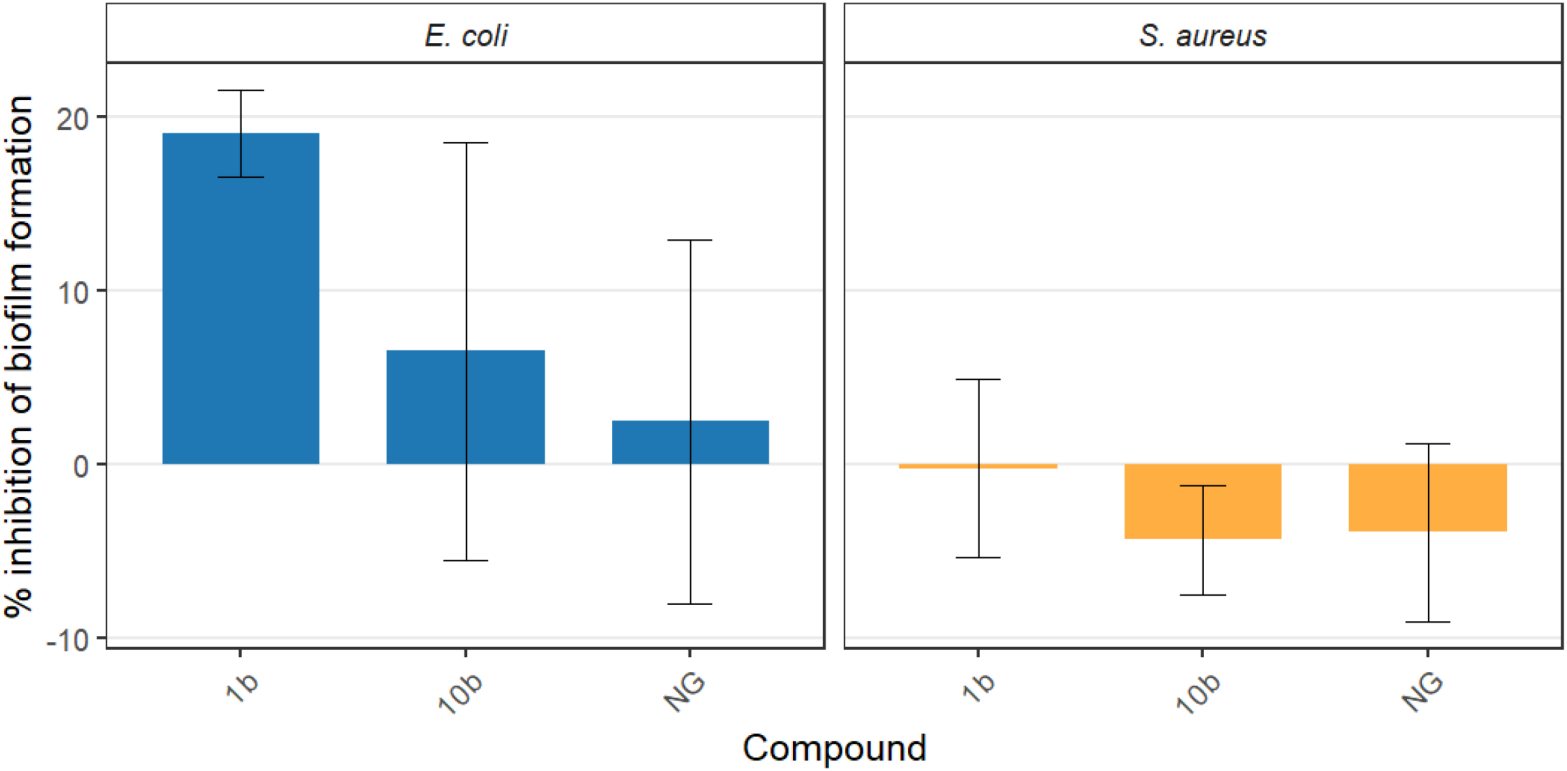
Comparison of the effectiveness of selected compounds in the eradication of *S. aureus* PCM 2054 or *E. coli* CCM 5712 biofilm from the catheter surface under flow conditions. NG – naringenin; 1b – 7-*O*-methylnaringenin oxime; 10b – 7-*O*-isopropylnaringenin oxime. Bars represent mean viability across replicates, and error bars denote standard deviation (SD).

The results obtained under flow-through conditions using a flow rate through the catheter lumen that reflects the physiological rate of final urine formation allowed for the reproduction of conditions present in a catheter following patient catheterization. At a concentration of 100µg mL^-1^, the tested compound exhibited antibacterial activity against the biofilm formed by *E. coli* CCM 5172. However, only one of them – the 1b derivative - caused a statistically significant reduction in biofilm structure, decreasing it by 17% compared with the control group (**Supplementary Tables 7**, **S8**). Interestingly, based on the previously obtained results concerning planktonic growth, viability, and antimicrobial activity, none of the tested compounds significantly affected the biofilm formed by *S. aureus* PCM 2054. Detailed results for all compounds against both strains used in the study are presented in **Figure 7**.

## 4 Discussion

The studies employed a complementary combination of methods for assessing antibacterial and anti-biofilm activity –static conditions, MIC/MBC assays, and a flow model – which allowed for a multidimensional analysis of the properties of the tested *O*-alkyl derivatives of NG and their oximes. The use of a single concentration in the biofilm assays reflects a screening-level approach and does not permit dose–response analysis of anti-biofilm activity. Among the tested compounds, oximes 1b and 10b were particularly noteworthy, as they imposed a strong reduction in planktonic growth and biofilm formation in the static model, especially in *S. aureus*. These compounds also achieved satisfactory MIC/MBC values, comparable for both the reference strain and MRSA strains, which is consistent with previous reports in the literature. Although numerous studies confirmed the broad spectrum of NG activity against *S. aureus*, with MIC values ranging from 0.6 to 62.5 µg mL⁻¹ (Parkar et al., 2008; Taechowisan et al., 2014; Mohammed et al., 2015; Ahmad et al., 2022), some reports indicated a lack of significant activity, with MIC values ranging from 0.256 to 16 mg mL⁻¹ (Dej-adisai et al., 2022; Ijoma et al., 2022). Structural modifications introduced to the NG molecule are definitely more promising. Kozłowska et al. showed that *O*-alkyl derivatives of NG achieved MIC values ranging from 6.25 to 50 µg mL⁻¹ against *S. aureus* ATCC 11632, while NG had an MIC value of 200 µg mL⁻¹ (Kozłowska et al., 2019). The activity of *O*-alkyl derivatives of NG and their oximes against the MRSA strain was also confirmed by studies conducted by Duda-Madej et al. The authors demonstrated activity at levels of 4–32 µg mL⁻¹ (Duda-Madej et al., 2020). Similar values were obtained for NG-pectin conjugates against *S. aureus* MTCC 7443 (NG: 6.5 µg mL⁻¹, conjugate: 2.5 µg mL⁻¹) (Mundlia et al., 2019). In turn, studies by Achika et al. and de Oliveira et al. confirmed the activity of NG and/or its glycosides against MSSA and MRSA (Achika et al., 2020; de Oliveira et al., 2022). These results indicate that appropriate structural modifications of NG can not only restore its activity against resistant pathogens, but also significantly increase it — even several times over. The results obtained in our study for compound 10b show that it exhibited the highest activity, achieving MIC/MBC values comparable to the most effective NG derivatives described in the literature (8–16 µg mL⁻¹). In turn, although oxime 1b also exceeded the activity of NG, its effect proved to be weaker than that of 10b. In addition, both compounds very effectively reduced the formation of *S. aureus* biofilm in the QTF model, which indicates a significant impact on the processes of adhesion and biofilm matrix formation.

NG and its derivatives have been at the center of research on natural compounds with antibacterial and anti-biofilm activity for several years. Numerous reports indicate that NG has a multidirectional effect on bacterial cells, inhibiting both planktonic growth and biofilm formation. It has been shown to reduce cell surface hydrophobicity, decrease exopolysaccharide production, and destabilize biofilm structure, which significantly impairs bacterial cell adhesion to the surface and their ability to form mature biofilm matrix. Wen et al. also demonstrated that NG modulated the expression of key genes regulating biofilm biogenesis in *S. aureus*, including *icaA, agrA, sarA, and sigB*, and activated pathways leading to the weakening of biofilm integrity, such as *cidA* and *dltB* (Wen et al., 2021). Thus, NG interferes with both the early stages of adhesion and the later stages of biofilm maturation, making it a promising candidate for therapeutic applications as a biofilm inhibitor. Our results confirm the multidirectional nature of NG and its derivatives. The apparent biofilm reduction observed for oximes in the QTF model occurred simultaneously with substantial inhibition of planktonic growth, particularly in *S. aureus*. Therefore, it cannot be unequivocally concluded whether the effect reflects a biofilm-specific mechanism or is predominantly secondary to antimicrobial activity.

In the flow model we used, only compound 1b showed statistically significant activity, and not against *S. aureus*, but against *E. coli* (17% decrease in viability). This difference between the previously obtained results in static conditions showing high efficacy of compounds 1b and 10b against *S. aureus* may be due to the fact that the flow model reflects more realistic physiological conditions. The limited activity of the tested compounds in our flow-through catheter model, despite their effectiveness in static assays, is consistent with previous reports showing a weak correlation between static and dynamic biofilm systems. Dai et al. reported no direct correspondence between these two models (Dai et al., 2017). Moreover, it has been shown that flow affects biofilm architecture, which in turn is associated with differential competition among bacterial strains (Nadell et al., 2017). Recent proteomic analyses by Huijboom et al. confirm this phenomenon, indicating that flow conditions, depending on the composition of the cell envelope, lead to changes in biofilm structure, contributing to its increased resistance (Huijboom et al., 2024). Therefore, the reduced activity observed in our flow-based model can be explained by several factors: 1) continuous medium exchange, which likely shortened the effective contact time and reduced the local compound concentration; 2) a more denser biofilm enriched in extracellular matrix, which may have limited the penetration of the compounds, and 3) adsorption of the substance to the catheter material and limited diffusion within biofilm structure, which may have reduced the effective dose reaching bacterial cells. These phenomena, in the form of EPS matrix complexity under flow conditions, leaching of test substances during flow, and a different cell phenotype in biofilm compared to planktonic forms, including changes in the expression of genes responsible for resistance and metabolism, the accumulation of which we observe in our studies, have been described previously (Wang et al; Zhang et al., 2013; Zhang et al., 2022). These mechanisms are consistent with numerous reports of increased resistance of biofilms formed under dynamic conditions (Zhang et al., 2022; Rashtchi et al., 2024). Static culture conditions typically favor higher estimated levels of anti-biofilm compound efficacy, as they lack shear forces and have limited mass transport. In contrast, flow models—which reflect real physiological conditions such as shear stress and continuous fluid flow—lead to the formation of more compact, resistant, and difficult-to-penetrate biofilms. The literature has repeatedly described cases in which strong activity observed under static conditions did not translate into activity in dynamic models, which is also an important interpretative context for our results (Wang et al; Van Der Veen and Abee, 2010; Klopper and Bester, 2024). These cumulative phenomena suggest that static conditions may overestimate anti-biofilm activity, whereas flow-based systems provide a more stringent and clinically relevant assessment of the efficacy of the tested compounds. In contrast to static conditions, where biofilm reduction closely paralleled planktonic growth inhibition, the limited antimicrobial activity observed in *E. coli* under flow suggests that the reduction in biofilm burden may reflect a more structure-specific effect rather than solely antimicrobial activity. This observation highlights the importance of dynamic models in distinguishing between antimicrobial and biofilm-related mechanisms.

The activity of NG against Gram-negative bacteria has already been demonstrated in the literature, although it depends on both the strain studied and the derivatives tested. This relationship is confirmed by numerous reports on various strains of *E. coli*, against which NG has shown different activity ranging from 0.5 to 8 mg mL⁻¹ (Mutheeswaran et al., 2017; Wang et al., 2018; de Oliveira et al., 2022; Yi et al., 2024). However, such a high MIC is not medically significant (Eumkeb and Chukrathok, 2013; Kozłowska and Duda-Madej, 2023). Recent studies show that derivatives of this compound have proven to be the most promising. To date, the following have been studied: i) *O*-alkyl derivatives of NG and their oximes exhibiting activity at MIC = 200 µg mL⁻¹ against *E. coli* ATCC 25922 (Kozłowska et al., 2019); ii) glucoside derivatives of NG (naringenin-7-*O*-β-glucopyranoside), for which an MIC of 50 µg mL⁻¹ against *E. coli* EHEC ATCC 05747 (Hernández-garcía et al., 2018); and conjugates of pure NG or its derivatives with pectin, showing activity ranging from 3.12 to 12.5 µg mL⁻¹ depending on the structural modification used (Mundlia et al., 2019; Achika et al., 2020). Confirmation that structural modifications of NG may be crucial for increasing its effectiveness against Gram-negative bacteria is provided by the study by Duda-Madej et al. The authors analyzed the activity of an expanded set of *O*-alkyl derivatives of NG and their oximes against ESBL^(+)^*E. coli* strains and showed that *O*-butyl, *O*-pentyl, and numerous NG oximes were active against these strains, achieving MICs of 16 to 64 µg mL⁻¹ and thus demonstrating significantly higher efficacy than free NG, which remained inactive against the tested strains (MIC >512 µg mL⁻¹) (Duda-Madej et al., 2020). Two of the same derivatives used in this study also showed very attractive activity against the ESBL^(+)^*E. coli* isolates tested in this urinary model, achieving MICs in the range of 8-32 µg mL⁻¹ vs. 64-128 µg mL⁻¹ for NG.

In light of the data provided by the literature, complemented by those presented in this article, NG stands out as a natural compound with a dual mechanism of action: it inhibits bacterial growth and effectively disrupts biofilm formation. Although its activity against *E. coli* is weaker than against *S. aureus*, it can be significantly increased through structural modifications, particularly through *O*-alkylation and oxime formation. These modifications significantly enhance the anti-biofilm properties and may be a key element in the action of these compounds, particularly in the context of eradicating biofilms of MDR strains of *S. aureus* and *E. coli*. In light of growing antibiotic resistance, NG and its derivatives represent a promising ground for further development of new antimicrobial molecules, while also appearing as promising compounds that could potentially enhance the effectiveness of therapies against biofilm pathogens and those that are difficult to eradicate. The results of our research provide evidence that targeted structural modifications of NG can significantly increase its effectiveness against both Gram-positive and Gram-negative pathogens, making it an attractive candidate for future preclinical studies. This is because they can enhance the action of NG against pathogens of chronic infections that show high tolerance to classical antibiotics.

### 4.1 Limit of the studies

It should be pointed out that this study was preliminary and focused only on evaluating the anti-biofilm activity of selected *O*-alkyl naringenin derivatives *in vitro*, under both static and flow conditions. Further studies should include the assessment of cytotoxicity and biocompatibility on urothelial cells, as well as a comparison of the efficacy of the tested compounds with commercial substances used in catheters. It will also be necessary to determine the stability of these compounds under physiological conditions, their release kinetics from polymeric materials, and their potential to include bacterial resistance during long-term exposure.

However, despite these limitations, the results obtained represent the first selective stage of promising chemical modifications within naringenin that improve anti-biofilm activity under conditions simulating the physiological environment of a urinary catheter. Our data may provide a basis for further, more advanced preclinical studies aimed at developing new strategies for preventing catheter-associated infections.

## 5 Conclusions

In summary, the experimental system employed in this study, combining static assays with a flow-based catheter model, enabled a stepwise evaluation of the anti-biofilm properties of *O*-alkyl naringenin derivatives under increasingly physiologically relevant conditions. While static models revealed pronounced antimicrobial and biofilm-reducing effects under static conditions (1b and 10b, particularly against *S. aureus*), these effects were substantially attenuated under dynamic flow conditions, highlighting the limited predictive value of static *in vitro* models when biofilm formation is assessed under dynamic conditions. Importantly, static biofilm models are known to overestimate compound efficacy due to the absence of shear stress, continuous nutrient exchange, and the complex architecture of mature biofilms formed under flow. The limited activity observed in the flow-through catheter model, therefore, highlights the need for cautious interpretation of translational potential and indicates that the results presented here should be regarded as an early-stage proof-of-concept rather than direct evidence of clinical applicability. Therefore, the observed biofilm reduction under static conditions should be interpreted as qualitative screening outcomes, potentially influenced by concurrent antimicrobial activity. Based on prior reports on naringenin, it is conceivable that naringenin oximes may affect biofilm architecture through mechanisms involving membrane-associated processes, EPS organization, or quorum sensing; however, these pathways were not addressed in the present study and should be validated in future mechanistic analyses. Future studies should therefore focus on comprehensive mechanistic analyses, evaluation of cytotoxicity and pharmacokinetic properties, and validation in appropriate *in vivo* models. Such an integrative approach will be essential to reliably assess the translational relevance of naringenin oxime derivatives as potential anti-biofilm agents, particularly in the context of catheter-associated infections caused by multidrug-resistant pathogens.

## Data Availability

The original contributions presented in the study are included in the article/**Supplementary Material**. Further inquiries can be directed to the corresponding author.
