# Supplementary material for: Gut-derived biofilm-forming bacteria as a source of catheter-associated infections: inhibitory effects of O-alkyl naringenin derivatives
Source: Front Cell Infect Microbiol. 2026 Mar 13;16:1768480. doi: 10.3389/fcimb.2026.1768480 (PMC13021772; doi:10.3389/fcimb.2026.1768480)
Supplement: Supplementary file 1 [file Table1.docx]

**Table S1.** Summary statistics of plankton growth inhibition.

| **Bacteria** | **Compound** | **Mean inhibition (%)** | **SD inhibition (%)** |
| --- | --- | --- | --- |
| *E. coli* | 1a | 18.51 | 7.82 |
| *E. coli* | 1b | 43.20 | 2.69 |
| *E. coli* | 2a | 11.62 | 4.28 |
| *E. coli* | 2b | 7.13 | 7.71 |
| *E. coli* | 4a | 16.01 | 5.29 |
| *E. coli* | 4b | 30.17 | 7.34 |
| *E. coli* | 10a | 1.39 | 5.11 |
| *E. coli* | 10b | 37.71 | 4.29 |
| *E. coli* | 12a | 27.53 | 0.89 |
| *E. coli* | 12b | 27.58 | 5.19 |
| *E. coli* | 14a | 23.45 | 6.22 |
| *E. coli* | 14b | 28.21 | 3.73 |
| *E. coli* | NG | 29.32 | 1.74 |
| *S. aureus* | 1a | 28.23 | 9.33 |
| *S. aureus* | 1b | 74.57 | 0.83 |
| *S. aureus* | 2a | –14.99 | 11.15 |
| *S. aureus* | 2b | –3.33 | 1.16 |
| *S. aureus* | 4a | 34.24 | 1.98 |
| *S. aureus* | 4b | 68.19 | 1.51 |
| *S. aureus* | 10a | 55.67 | 3.96 |
| *S. aureus* | 10b | 69.01 | 0.43 |
| *S. aureus* | 11a | 14.79 | 4.19 |
| *S. aureus* | 12a | 65.53 | 2.10 |
| *S. aureus* | 12b | 70.74 | 0.42 |
| *S. aureus* | 14a | 53.19 | 3.25 |
| *S. aureus* | 14b | 72.73 | 1.67 |
| *S. aureus* | NG | 27.84 | 5.19 |

**Table S2.** Results of one-sided Wilcoxon rank-sum tests evaluating whether each compound reduced plankton growth relative to the DMSO control. Comparisons were performed separately for each bacterial species (*E. coli*, *S. aureus*) and experimental set (A, B). For each DMSO–compound pair, the table reports sample sizes (n1, n2), test statistic, raw p-values, and Benjamini–Hochberg–corrected p-values (p_bh_). Significance after FDR correction is indicated as * (0.01 ≤ *p_bh_* < 0.05), ** (0.001 ≤ *p_bh_* < 0.01), *** (*p_bh_* < 0.001), or ns (*p_bh_* ≥ 0.05).

| **Bacteria** | **Set** | **Group 1** | **Group 2** | **n_1_** | **n_2_** | **statistic** | **p_raw_** | ***p_bh_*** | **Signif. *p_bh_*** |
| --- | --- | --- | --- | --- | --- | --- | --- | --- | --- |
| *E.coli*  *E.coli*  *E.coli*  *E.coli*  *E.coli*  *E.coli*  *E.coli*  *E.coli*  *E.coli*  *E.coli*  *E.coli*  *E.coli*  *E.coli* | A | DMSO | 10b | 3 | 4 | 12 | 0.026 | 0.02600000 | * |
|  | A | DMSO | 12a | 3 | 4 | 12 | 0.026 | 0.02600000 | * |
|  | A | DMSO | 12b | 3 | 4 | 12 | 0.026 | 0.02600000 | * |
|  | A | DMSO | 1a | 3 | 4 | 12 | 0.026 | 0.02600000 | * |
|  | A | DMSO | 1b | 3 | 4 | 12 | 0.026 | 0.02600000 | * |
|  | A | DMSO | 4b | 3 | 4 | 12 | 0.026 | 0.02600000 | * |
|  | A | DMSO | NG | 3 | 4 | 12 | 0.025 | 0.02600000 | * |
|  | B | DMSO | 14a | 3 | 4 | 12 | 0.026 | 0.03900000 | * |
|  | B | DMSO | 14b | 3 | 4 | 12 | 0.026 | 0.03900000 | * |
|  | B | DMSO | 2a | 3 | 4 | 12 | 0.026 | 0.03900000 | * |
|  | B | DMSO | 4a | 3 | 4 | 12 | 0.026 | 0.03900000 | * |
|  | B | DMSO | 2b | 3 | 4 | 9 | 0.188 | 0.22560000 | ns |
|  | B | DMSO | 10a | 3 | 4 | 7 | 0.430 | 0.43000000 | ns |
| *S.aureus* | A | DMSO | 10a | 3 | 4 | 12 | 0.026 | 0.03466667 | * |
| *S.aureus* | A | DMSO | 11a | 3 | 4 | 12 | 0.026 | 0.03466667 | * |
| *S.aureus* | A | DMSO | 12b | 3 | 4 | 12 | 0.026 | 0.03466667 | * |
| *S.aureus* | A | DMSO | 14b | 3 | 4 | 12 | 0.026 | 0.03466667 | * |
| *S.aureus* | A | DMSO | 1a | 3 | 4 | 12 | 0.026 | 0.03466667 | * |
| *S.aureus* | A | DMSO | 4a | 3 | 4 | 12 | 0.026 | 0.03466667 | * |
| *S.aureus* | A | DMSO | 2a | 3 | 4 | 1 | 0.974 | 0.97400000 | ns |
| *S.aureus* | A | DMSO | 2b | 3 | 4 | 3 | 0.894 | 0.97400000 | ns |
| *S.aureus* | B | DMSO | 10b | 4 | 4 | 16 | 0.015 | 0.01500000 | * |
| *S.aureus* | B | DMSO | 12a | 4 | 4 | 16 | 0.015 | 0.01500000 | * |
| *S.aureus* | B | DMSO | 14a | 4 | 4 | 16 | 0.015 | 0.01500000 | * |
| *S.aureus* | B | DMSO | 1b | 4 | 4 | 16 | 0.015 | 0.01500000 | * |
| *S.aureus* | B | DMSO | 4b | 4 | 4 | 16 | 0.015 | 0.01500000 | * |
| *S.aureus* | B | DMSO | NG | 4 | 4 | 16 | 0.015 | 0.01500000 | * |

**Table S3.** **Results of one-sided Wilcoxon rank-sum tests evaluating whether each compound reduced planktonic cell viability (MTT assay) relative to the DMSO control.** Comparisons were performed separately for each bacterial species (E. coli, S. aureus) and experimental set (A, B). For each DMSO–compound pair, the table reports the sample sizes (n1, n2), test statistic, raw p-values, and Benjamini–Hochberg–corrected p-values (pbh). Statistical significance after FDR correction is indicated as * (0.01 ≤ *p_bh_* < 0.05), ** (0.001 ≤ *p_bh_* < 0.01), *** (*p_bh_* < 0.001), or ns (*p_bh_* ≥ 0.05).

| **Bacteria** | **Set** | **Group 1** | **Group 2** | **n_1_** | **n_2_** | **statistic** | **p_raw_** | ***p_bh_*** | **Signif. *p_bh_*** |
| --- | --- | --- | --- | --- | --- | --- | --- | --- | --- |
| *E.coli* | A | DMSO | 10b | 3 | 4 | 12.0 | 0.026 | 0.09100000 | ns |
| *E.coli* | A | DMSO | 1b | 3 | 4 | 12.0 | 0.026 | 0.09100000 | ns |
| *E.coli* | A | DMSO | NG | 3 | 4 | 10.0 | 0.108 | 0.25200000 | ns |
| *E.coli* | A | DMSO | 12b | 3 | 4 | 8.0 | 0.298 | 0.52150000 | ns |
| *E.coli* | A | DMSO | 4b | 3 | 4 | 6.0 | 0.570 | 0.79800000 | ns |
| *E.coli* | A | DMSO | 12a | 3 | 4 | 2.0 | 0.944 | 0.94400000 | ns |
| *E.coli* | A | DMSO | 1a | 3 | 4 | 3.0 | 0.892 | 0.94400000 | ns |
| *E.coli* | B | DMSO | 10a | 4 | 4 | 16.0 | 0.015 | 0.10500000 | ns |
| *E.coli* | B | DMSO | 14b | 4 | 4 | 15.0 | 0.030 | 0.10500000 | ns |
| *E.coli* | B | DMSO | 11a | 4 | 4 | 13.0 | 0.097 | 0.22633333 | ns |
| *E.coli* | B | DMSO | 2a | 4 | 4 | 12.0 | 0.156 | 0.27300000 | ns |
| *E.coli* | B | DMSO | 14a | 4 | 4 | 9.0 | 0.443 | 0.51683333 | ns |
| *E.coli* | B | DMSO | 2b | 4 | 4 | 9.5 | 0.386 | 0.51683333 | ns |
| *E.coli* | B | DMSO | 4a | 4 | 4 | 6.0 | 0.765 | 0.76500000 | ns |
| *S.aureus* | A | DMSO | 10a | 3 | 4 | 12.0 | 0.026 | 0.02971429 | * |
| *S.aureus* | A | DMSO | 11a | 3 | 4 | 12.0 | 0.025 | 0.02971429 | * |
| *S.aureus* | A | DMSO | 12b | 3 | 4 | 12.0 | 0.026 | 0.02971429 | * |
| *S.aureus* | A | DMSO | 14b | 3 | 4 | 12.0 | 0.025 | 0.02971429 | * |
| *S.aureus* | A | DMSO | 1a | 3 | 4 | 12.0 | 0.026 | 0.02971429 | * |
| *S.aureus* | A | DMSO | 2b | 3 | 4 | 12.0 | 0.026 | 0.02971429 | * |
| *S.aureus* | A | DMSO | 4a | 3 | 4 | 12.0 | 0.026 | 0.02971429 | * |
| *S.aureus* | A | DMSO | 2a | 3 | 4 | 4.0 | 0.812 | 0.81200000 | ns |
| *S.aureus* | B | DMSO | 10b | 4 | 4 | 16.0 | 0.015 | 0.01800000 | * |
| *S.aureus* | B | DMSO | 12a | 4 | 4 | 16.0 | 0.015 | 0.01800000 | * |
| *S.aureus* | B | DMSO | 14a | 4 | 4 | 16.0 | 0.015 | 0.01800000 | * |
| *S.aureus* | B | DMSO | 1b | 4 | 4 | 16.0 | 0.015 | 0.01800000 | * |
| *S.aureus* | B | DMSO | 4b | 4 | 4 | 16.0 | 0.015 | 0.01800000 | * |
| *S.aureus* | B | DMSO | NG | 4 | 4 | 13.0 | 0.097 | 0.09700000 | ns |

**Table S4.** Summary statistics of planktonic viability measured by the MTT assay.

| **Bacteria** | **Compound** | **Mean viability (%)** | **SD viability (%)** |
| --- | --- | --- | --- |
| *E. coli* | 1a | 104.39 | 4.45 |
| *E. coli* | 1b | 79.69 | 3.43 |
| *E. coli* | 2a | 91.10 | 7.59 |
| *E. coli* | 2b | 98.04 | 15.72 |
| *E. coli* | 4a | 107.23 | 11.22 |
| *E. coli* | 4b | 98.25 | 2.16 |
| *E. coli* | 10a | 80.46 | 3.66 |
| *E. coli* | 10b | 81.64 | 5.10 |
| *E. coli* | 11a | 85.83 | 9.77 |
| *E. coli* | 12a | 109.23 | 8.22 |
| *E. coli* | 12b | 96.19 | 4.24 |
| *E. coli* | 14a | 96.33 | 10.51 |
| *E. coli* | 14b | 86.09 | 3.39 |
| *E. coli* | NG | 91.22 | 6.88 |
| *S. aureus* | 1a | 80.36 | 4.33 |
| *S. aureus* | 1b | 21.70 | 2.78 |
| *S. aureus* | 2a | 109.32 | 16.08 |
| *S. aureus* | 2b | 78.55 | 8.16 |
| *S. aureus* | 4a | 63.35 | 0.66 |
| *S. aureus* | 4b | 36.75 | 1.85 |
| *S. aureus* | 10a | 34.14 | 1.74 |
| *S. aureus* | 10b | 23.19 | 2.53 |
| *S. aureus* | 11a | 54.57 | 1.33 |
| *S. aureus* | 12a | 32.09 | 0.41 |
| *S. aureus* | 12b | 45.16 | 3.72 |
| *S. aureus* | 14a | 34.30 | 0.34 |
| *S. aureus* | 14b | 34.57 | 0.80 |
| *S. aureus* | NG | 91.02 | 3.79 |

**Table S5.** Summary statistics of inhibition of biofilm formation measured by the QTF assay.

| **Bacteria** | **Compound** | **Mean inhibition (%)** | **SD viability (%)** |
| --- | --- | --- | --- |
| *E. coli* | 1a | 5.681818 | 5.410173 |
| *E. coli* | 1b | 22.15909 | 3.881421 |
| *E. coli* | 2a | -37.14286 | 5.216405 |
| *E. coli* | 2b | -17.14286 | 2.857143 |
| *E. coli* | 4a | -38.57143 | 11.547005 |
| *E. coli* | 4b | 29.54545 | 6.428243 |
| *E. coli* | 10a | 6.428571 | 4.879500 |
| *E. coli* | 10b | 3.409091 | 6.013071 |
| *E. coli* | 12b | 23.86364 | 4.351941 |
| *E. coli* | 14a | 27.14286 | 7.190319 |
| *E. coli* | 14b | 7.142857 | 4.948717 |
| *E. coli* | NG | 35.79545 | 5.039445 |
| *S. aureus* | 1a | -35.00000 | 23.372174 |
| *S. aureus* | 1b | 36.31285 | 5.319500 |
| *S. aureus* | 2a | -29.52381 | 13.502330 |
| *S. aureus* | 2b | -31.42857 | 7.377111 |
| *S. aureus* | 4a | -27.61905 | 8.728716 |
| *S. aureus* | 4b | -15.08380 | 10.560482 |
| *S. aureus* | 10a | 45.71429 | 5.216405 |
| *S. aureus* | 10b | 59.77654 | 4.827367 |
| *S. aureus* | 12b | -27.85714 | 5.890151 |
| *S. aureus* | 14a | -16.75978 | 11.154547 |
| *S. aureus* | 14b | -43.57143 | 20.914468 |
| *S. aureus* | NG | 10.61453 | 9.122867 |

**Table S6. Results of one-sided Wilcoxon rank-sum tests evaluating whether each compound reduced biofilm formation (QFT assay) relative to the DMSO control.** Comparisons were performed separately for each bacterial species (E. coli, S. aureus) and experimental set (A, B). For each DMSO–compound pair, the table reports the sample sizes (n1, n2), test statistic, raw p-values, and Benjamini–Hochberg–corrected p-values (pbh). Statistical significance after FDR correction is indicated as * (0.01 ≤ *p_bh_* < 0.05), ** (0.001 ≤ *p_bh_* < 0.01), *** (*p_bh_* < 0.001), or ns (*p_bh_* ≥ 0.05).

| Bacteria | Set | Group 1 | Group 2 | n1 | n2 | statistic | p_raw_ | p_bh_ | Signif. p_bh_ |
| --- | --- | --- | --- | --- | --- | --- | --- | --- | --- |
| *E. coli* | A | DMSO | 12b | 3 | 4 | 12.0 | 0.025 | 0.0390 | * |
| *E. coli* | A | DMSO | 1b | 3 | 4 | 12.0 | 0.026 | 0.0390 | * |
| *E. coli* | A | DMSO | 4b | 3 | 4 | 12.0 | 0.025 | 0.0390 | * |
| *E. coli* | A | DMSO | NG | 3 | 4 | 12.0 | 0.025 | 0.0390 | * |
| *E. coli* | A | DMSO | 1a | 3 | 4 | 6.05 | 0.500 | 0.6000 | ns |
| *E. coli* | A | DMSO | 10b | 3 | 4 | 5.05 | 0.639 | 0.6390 | ns |
| *E. coli* | B | DMSO | 14a | 3 | 4 | 12.0 | 0.025 | 0.1500 | ns |
| *E. coli* | B | DMSO | 14b | 3 | 4 | 8.05 | 0.236 | 0.7080 | ns |
| *E. coli* | B | DMSO | 10a | 3 | 4 | 7.05 | 0.361 | 0.7220 | ns |
| *E. coli* | B | DMSO | 2a | 3 | 4 | 0.0 | 0.989 | 0.9890 | ns |
| *E. coli* | B | DMSO | 2b | 3 | 3 | 0.5 | 0.977 | 0.9890 | ns |
| *E. coli* | B | DMSO | 4a | 3 | 4 | 0.0 | 0.989 | 0.9890 | ns |
| *S. aureus* | A | DMSO | 10a | 3 | 4 | 12.0 | 0.026 | 0.1820 | ns |
| *S. aureus* | A | DMSO | 12b | 3 | 4 | 3.05 | 0.860 | 0.9740 | ns |
| *S. aureus* | A | DMSO | 14b | 3 | 4 | 1.0 | 0.974 | 0.9740 | ns |
| *S. aureus* | A | DMSO | 1a | 3 | 4 | 1.05 | 0.963 | 0.9740 | ns |
| *S. aureus* | A | DMSO | 2a | 3 | 3 | 1.05 | 0.939 | 0.9740 | ns |
| *S. aureus* | A | DMSO | 2b | 3 | 4 | 2.05 | 0.923 | 0.9740 | ns |
| *S. aureus* | A | DMSO | 4a | 3 | 3 | 2.0 | 0.905 | 0.9740 | ns |
| *S. aureus* | B | DMSO | 10b | 4 | 4 | 16.0 | 0.015 | 0.0375 | * |
| *S. aureus* | B | DMSO | 1b | 4 | 4 | 16.0 | 0.015 | 0.0375 | * |
| *S. aureus* | B | DMSO | NG | 4 | 4 | 12.0 | 0.156 | 0.2600 | ns |
| *S. aureus* | B | DMSO | 14a | 4 | 4 | 2.05 | 0.959 | 0.9590 | ns |
| *S. aureus* | B | DMSO | 4b | 4 | 4 | 3.05 | 0.932 | 0.9590 | ns |
|  |  |  |  |  |  |  |  |  |  |

**Table S7.** Summary statistics of biofilm inhibition.

| **Bacteria** | **Compound** | **Mean inhibition (%)** | **SD viability (%)** |
| --- | --- | --- | --- |
| *E. coli* | 1b | 19.08631 | 2.514459 |
| *E. coli* | 10b | 6.513053 | 12.016762 |
| *E. coli* | NG | 2.477357 | 10.475980 |
| *S. aureus* | 1b | -0.2411576 | 5.133102 |
| *S. aureus* | 10b | -4.335094 | 3.152556 |
| *S. aureus* | NG | -3.938907 | 5.176117 |

**Table S8. Results of one-sided Wilcoxon rank-sum tests evaluating biofilm inhibition relative to the control.** Comparisons were performed separately for each bacterial species E. coli, S. aureus. For each control–compound pair, the table reports the sample sizes (n1, n2), test statistic, raw p-values, and Benjamini–Hochberg–corrected p-values (pbh). Statistical significance after FDR correction is indicated as * (0.01 ≤ *p_bh_* < 0.05), ** (0.001 ≤ *p_bh_* < 0.01), *** (*p_bh_* < 0.001), or ns (*p_bh_* ≥ 0.05).

| **Bacteria** | **Group 1** | **Group 2** | **n_1_** | **n_2_** | **statistic** | **p_raw_** | ***p_bh_*** | **Signif. *p_bh_*** |
| --- | --- | --- | --- | --- | --- | --- | --- | --- |
| E.coli | DMSO | 1b | 9 | 8 | 71.5 | 0.000373 | 0.001119 | ** |
| E.coli | DMSO | 10b | 9 | 9 | 56.0 | 0.092000 | 0.138000 | ns |
| E.coli | DMSO | NG | 9 | 9 | 47.0 | 0.298000 | 0.298000 | ns |
| S.aureus | DMSO | 10b | 9 | 7 | 19.5 | 0.907000 | 0.907000 | ns |
| S.aureus | DMSO | 1b | 9 | 9 | 38.0 | 0.605000 | 0.907000 | ns |
| S.aureus | DMSO | NG | 9 | 9 | 28.0 | 0.875000 | 0.907000 | ns |
